# SEC61: a potential therapeutic target in transplantation

**DOI:** 10.3389/fimmu.2026.1787920

**Published:** 2026-03-18

**Authors:** Maria Laura Saiz, Cristian Ruiz Bernet, Carlos Lopez-Larrea, Beatriz Suarez-Alvarez

**Affiliations:** 1 Translational Immunology, Health Research Institute of the Principality of Asturias (ISPA), Oviedo, Spain; 2 Kidney Disease Research Network, RICORS2040, Instituto de Salud Carlos III (ISCIII), Madrid, Spain

**Keywords:** alloimmunity, ER proteostasis, ER stress, immunosuppression, ischemia–reperfusion injury, kidney transplantation, Sec61, translocon

## Abstract

The SEC61 translocon complex has emerged as a multifunctional therapeutic target linking protein secretion, calcium homeostasis, and immune regulation in kidney transplantation. Beyond canonical protein translocation, SEC61 regulates antigen cross-presentation, cytokine secretion (IL-2, IFN-γ, TNF-α), surface activation molecules (CD62L), and functions as an endoplasmic reticulum calcium-leak channel that modulates unfolded protein response activation under specific physiological and stress conditions. During ischemia–reperfusion injury, ATP depletion impairs SERCA-mediated calcium reuptake while SEC61-mediated calcium efflux persists, triggering ER stress and tubular injury. Selective pharmacological SEC61 inhibition has been proposed to confer multiple immunomodulatory and cytoprotective effects - including reduced antigen cross-presentation, suppression of high-burden secretory lymphocytes, limited T cell migration, and intrinsic antiviral activity through blockade of envelope glycoprotein biogenesis—based on mechanistic and preclinical evidence, although these effects remain to be validated in transplant-specific models. Emerging phase I oncology data with client-selective inhibitors demonstrate the feasibility of pharmacologic SEC61 modulation in humans, although the safety, dosing, and patient population in transplantation may differ substantially from oncology settings. This review examines SEC61’s multifaceted roles in transplant immunobiology and its therapeutic potential as a novel immunomodulatory target in kidney transplantation.

## Introduction

Kidney transplantation remains the optimal therapeutic approach for end-stage renal disease (ESRD), yet persistent challenges including ischemia–reperfusion injury (IRI), delayed graft function, and alloimmune rejection continue to limit graft survival and long-term patient outcomes (1–3). Acute cellular and antibody-mediated rejection episodes affect approximately 10–15% of transplant recipients during the first postoperative year, while chronic antibody-mediated rejection continues to represent a leading cause of late transplant failure. Current immunosuppressive regimens, although effective in reducing acute rejection rates, have substantial adverse effects including dose-dependent nephrotoxicity, enhanced susceptibility to opportunistic infections and malignancies, and metabolic complications that compromise long-term patient health. The SEC61 translocon complex, long recognized primarily for facilitating nascent protein import across the endoplasmic reticulum (ER) membrane (4, 5), has recently emerged as a multifunctional cellular regulator with substantial implications for transplantation biology. Beyond its canonical role in protein translocation, SEC61 has been implicated in ER calcium efflux that is dynamically regulated in a context-dependent manner during cellular stress conditions (6, 7), participates in unfolded protein response (UPR) regulation (8), and influences diverse immune homeostatic pathways (9, 10). This heterotrimer, comprising SEC61α, SEC61β, and SEC61γ subunits, functionally integrates multiple cellular stress responses that collectively determine transplant graft outcomes. Selective pharmacological inhibition of SEC61 produces potent immunosuppressive effects demonstrated across multiple experimental model systems, although predominantly in *in vitro* systems and non-transplant *in vivo* models (10, 11), and *SEC61A1* mutations cause heritable kidney disease, establishing a direct mechanistic connection between SEC61 dysfunction and progressive renal pathology (12, 13). This review examines SEC61 detailed involvement in immune regulation and IRI pathophysiology, presents clinical evidence from emerging inhibitor candidates including phase I data from KZR-261 (14, 15), and proposes a rational framework for SEC61-targeted immunosuppression in kidney transplantation. The following sections address SEC61 structure, mechanism, and links to the unfolded protein response; its role in renal ischemia–reperfusion injury; its involvement in alloimmune responses, including cross-presentation, cytokine secretion, and lymphocyte trafficking; the antiviral potential of SEC61 inhibition and lessons from oncology trials; and finally, tubular preservation strategies and a conceptual framework for clinical translation.

## SEC61 structure, mechanism and link to UPR

The SEC61 complex is the core protein-conducting channel of the ER, responsible for co- and post-translational translocation of secretory and membrane proteins. SEC61α forms a ten-transmembrane “clam-shell” architecture with a central aqueous pore and a lateral gate that recognizes signal peptides and transmembrane helices, while SEC61β and SEC61γ stabilize the complex and recruit accessory factors (4–6). Beyond protein import, SEC61 functions as an atypical Ca^2+^ leak channel that continuously allows efflux of Ca^2+^ from the ER lumen to the cytosol. This leak is not constitutive but is regulated by BiP binding and cellular stress conditions and normally counterbalanced by sarco/endoplasmic reticulum Ca^2+^-ATPases (SERCA), which pump Ca^2+^ back into the ER and maintain a steep luminal–cytosolic gradient (6, 9). BiP binds to SEC61 and helps maintain the pore in a closed state, limiting Ca^2+^ leakage under resting conditions. Because many ER chaperones and folding enzymes are Ca^2+^-dependent, disruption of the SEC61–SERCA–BiP balance can impair folding capacity and contribute to ER stress (7, 8). The UPR is activated when misfolded proteins accumulate in the ER, engaging the stress sensors IRE1, PERK, and ATF6. In its adaptive phase, the UPR reduces new protein synthesis, increases expression of chaperones and ER-associated degradation (ERAD) components, and attempts to restore proteostasis. If stress is severe or prolonged, signaling shifts toward a terminal program characterized by CHOP induction and activation of pro-apoptotic pathways (16). SEC61 lies at the crossroads of these processes: it is required for influx of new client proteins into the ER and contributes to retrotranslocation of misfolded proteins destined for degradation (4, 9, 10), although other pathways involving Derlin and Hrd1 complexes also participate in ERAD substrate extraction (17).

Structural and chemical biology studies have shown that small molecules can modulate SEC61 in a relatively client-selective manner (11, 18), with the spectrum of affected substrates depending on inhibitor chemistry and cellular context. For instance, cotransin-class SEC61 inhibitors like KZR-8445 exhibit client selectivity through signal peptide mimicry, where the inhibitor’s binding pose is dynamically shaped by the substrate’s signal sequence properties—such as hydrophobicity and charge—enabling potent blockade of pro-inflammatory cytokine translocation while sparing housekeeping secretion (11). These advances thus support scaffold optimization of natural product SEC61 inhibitors for therapeutic precision, targeting secretory cargoes (e.g., cytokines, viral glycoproteins) with minimal disruption to basal ER traffic, analogous to cotransin signal peptide discrimination (18, 19).

## SEC61 in renal ischemia-reperfusion

Ischemia–reperfusion injury is an inevitable insult in kidney transplantation and a major determinant of delayed graft function and early graft dysfunction (1, 2). IRI is a complex, multifactorial process involving oxidative stress, inflammation, mitochondrial dysfunction, and ER stress, among which SEC61-mediated mechanisms might represent one of several contributing pathways. During ischemia, ATP depletion impairs SERCA activity, preventing efficient reuptake of Ca^2+^ into the ER lumen (20). In contrast, SEC61-mediated Ca^2+^ leak persists, leading to progressive depletion of luminal Ca^2+^ and cytosolic Ca^2+^ overload (21). This disturbed Ca^2+^ homeostasis favors mitochondrial Ca^2+^ accumulation, opening of the mitochondrial permeability transition pore and activation of cell death pathways (9). Reperfusion reintroduces oxygen, triggering a burst of reactive oxygen species (ROS) that further damage ER and mitochondrial membranes and aggravate Ca^2+^ dysregulation. At the same time, protein synthesis resumes, and SEC61 channels newly synthesized polypeptides into an ER compartment already stressed by Ca^2+^ depletion and oxidative injury. The combined burden of misfolded proteins, low luminal Ca^2+^ and ROS potently activates the UPR via IRE1, PERK and ATF6. Within this sequence, SEC61 occupies an ambivalent position. Continued protein influx through SEC61 during very early reperfusion may exacerbate ER stress by increasing folding demand. However, SEC61-dependent translocation is also required for synthesis of chaperones and ERAD components necessary to resolve stress. A strong SEC61 blockade during the immediate reperfusion window could reduce new client influx but simultaneously interfere with adaptive UPR programs, tipping the balance toward terminal UPR and tubular apoptosis (8, 9). Clinically, this reasoning argues for delaying SEC61-targeted therapy until approximately postoperative day 1, as this approach avoids disrupting critical early reperfusion repair responses. However, because many of the aforementioned mechanisms have primarily been investigated in myocardial infarction models, their relevance must be specifically established in renal pathology. Accordingly, this timing proposal remains a mechanistically rational hypothesis that requires validation through dedicated preclinical experiments, such as testing different SEC61 inhibitor start times and durations in murine kidney transplant models incorporating ischemia–reperfusion injury (e.g., fully MHC-mismatched orthotopic transplants with defined warm/cold ischemia times), with endpoints including tubular injury markers, alloimmune activation (T cell infiltration, cytokine profiles), and graft function.

## SEC61 in alloimmune responses: cross-presentation, cytokine secretion, and lymphocyte trafficking

After the graft has survived the initial IRI phase, alloimmune mechanisms become the predominant threat to graft survival. SEC61 functionally integrates multiple critical steps in this cascade: antigen presentation, T and B cell activation, cytokine production, and leukocyte trafficking (10, 22–26). Cross-presentation of donor-derived antigens on MHC class I molecules by recipient dendritic cells is central to priming cytotoxic CD8^+^ T cells against the allograft (27). In this pathway, exogenous antigens internalized into endosomes are translocated into the cytosol, processed by the proteasome, and loaded onto MHC class I molecules. SEC61 has been proposed to form the channel that moves internalized antigens from endosomes into the cytosol, enabling proteasome-dependent cross-presentation on MHC I. Data from Zehner et al. (22) support this model, showing that when SEC61 is prevented from reaching endosomes, antigen export to the cytosol and cross-presentation are specifically blocked, while other antigen presentation routes remain intact. In contrast, Grotzke et al. (24) show that acute pharmacological SEC61 inhibition does not immediately impair antigen export or cross-presentation, and that defects arise only after prolonged blockade, when many SEC61-dependent proteins (including MHC I/II) are lost; this suggests that sustained SEC61 inhibition mainly disrupts cross-presentation indirectly by depleting the antigen-presenting machinery rather than by acutely blocking an endosomal export pore. Thus, while the precise molecular mechanism remains under investigation—whether SEC61 acts directly as an endosomal export channel or indirectly by maintaining the antigen-presenting machinery—both models suggest that SEC61 function may be important for efficient cross-presentation. Consistently, interfering with SEC61 function ultimately compromises this process, underscoring it as a potentially relevant and pharmacologically sensitive checkpoint in CD8^+^ T cell priming. This raises the possibility that selective SEC61 inhibition in the early post-transplant period could substantially reduce the magnitude of CD8^+^ T cell responses to allogeneic donor antigens, a hypothesis that still requires validation in transplant-specific models before therapeutic extrapolation.

SEC61 is also indispensable for cytokine secretion and surface expression of T cell activation receptors. Canonical pro-inflammatory cytokines such as IL-2, IFN-γ and TNF-α all possess signal peptides that route them through SEC61 into the ER and along the secretory pathway (10, 11, 26, 28). These cytokines are critical for alloimmune responses: IL-2 drives T cell proliferation; IFN-γ activates macrophages and endothelial cells and TNF-α amplifies local inflammation. Beyond secreted cytokines, surface molecules involved in T cell activation such as L-selectin (CD62L)— require SEC61-dependent translocation for proper biogenesis and trafficking to the plasma membrane (10). Moreover, monocyte-derived cytokine secretion is profoundly inhibited by the SEC61 inhibitor mycolactone, which prevents the translation of key inflammatory mediators including TNF, IL-1β, IL-6, IL-8, IL-10, IP-10, and COX-2 (25). Controlling monocyte-derived inflammatory responses is critical in allograft rejection because excessive monocyte activation amplifies tissue-damaging cytokine cascades, accelerates graft injury, and compromises long-term transplant survival (29). It should be noted that mycolactone exhibits broad cytotoxicity (30), and its effects on myeloid cells may not fully generalize to more selective SEC61 inhibitors currently in development. However, mycolactone also widely inhibits cytokine production in both T cells and myeloid cells, supporting its characterization as a more potent and less selective SEC61 inhibitor than other SEC61 inhibitors (10).

Leukocyte trafficking into the graft constitutes a third critical level at which SEC61 plays a role. Leukocyte adhesion molecules depend on SEC61 for insertion into the ER and subsequent surface expression (31). Endothelial cell adhesion molecules (CAMs) such as VCAM-1, ICAM-1, and E-selectin are key regulators of leukocyte migration during inflammation. Inhibitors of the SEC61 translocon, including HUN-7293, CAM741, and cotransin, selectively block the ER translocation of these molecules and other secretory proteins like TNFα and VEGF (32, 33). By preventing proper CAM expression, SEC61 inhibition effectively reduces endothelial adhesion and represents a potential strategy to modulate immune responses and inflammation. However, SEC61 inhibition has also been associated with altered morphology and barrier function of endothelial cells due to the depletion of junctional proteins such as VE-cadherin, TIE-1, TIE-2 and JAM-C. These proteins are all SEC61-dependent and require co-translational translocation into the ER lumen to complete their biosynthesis (34). Thus, in the transplant setting, SEC61 inhibition may exert a dual potentially opposing effect: while it can limit leukocyte recruitment by suppressing CAM and cytokine expression, the concomitant disruption of endothelial junctions could paradoxically increase vascular permeability and compromise graft microvascular integrity. The net effect on graft inflammation is therefore context-dependent, and systematic experimental studies—including assessments of endothelial barrier function, vascular leak, and inflammatory infiltrates—will be essential to determine whether beneficial or harmful effects predominate *in vivo*.

## Antiviral “dual-action” potential of SEC61 inhibition

**Figure 1:**
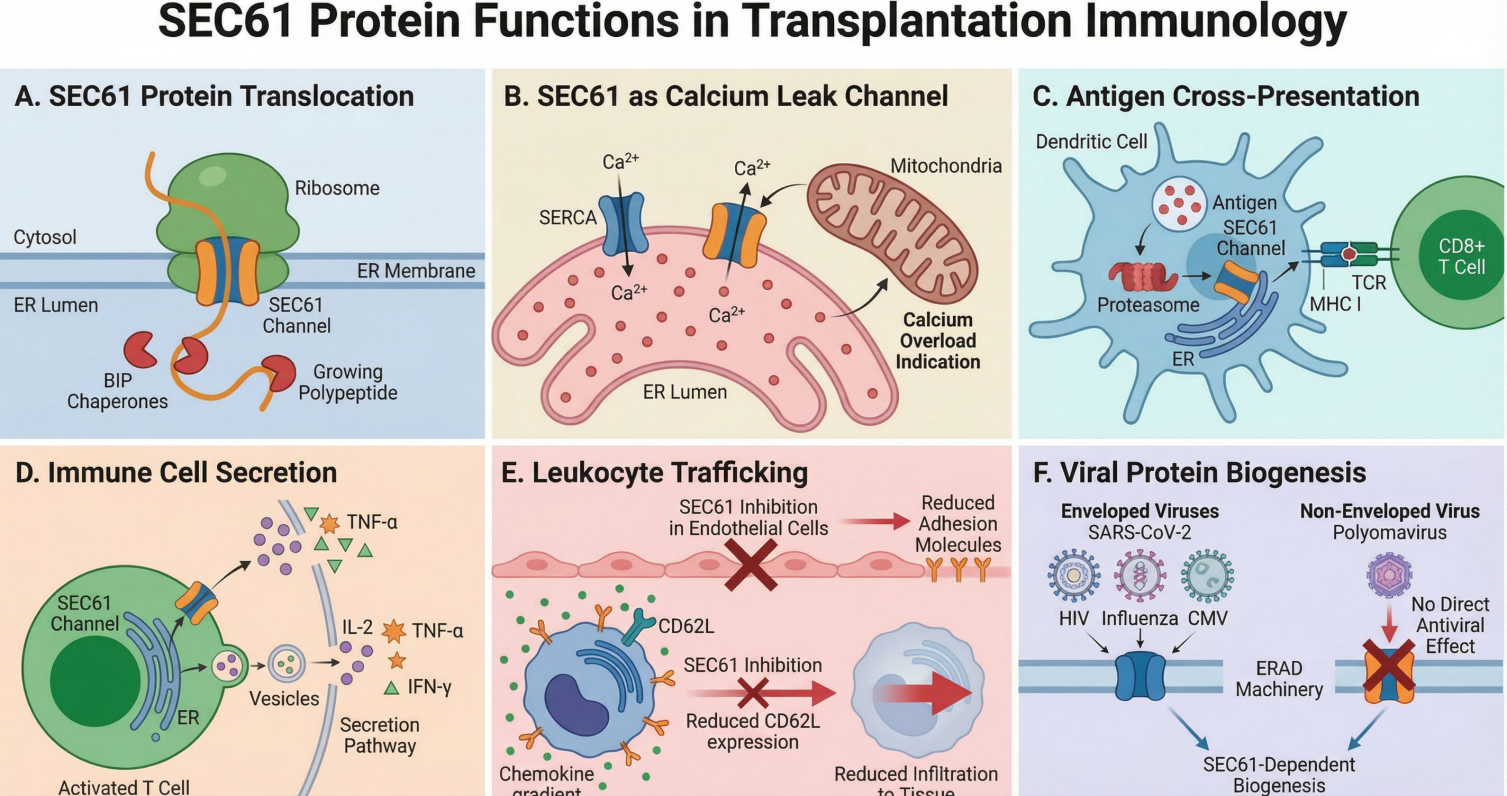
Multifaceted mechanisms of SEC61 inhibition in kidney transplantation. This comprehensive schematic illustrates the central role of the SEC61 channel in cellular physiology and transplant-related pathology. **(A)** Protein Translocation: SEC61 forms the core translocon complex for nascent polypeptides entering the endoplasmic reticulum (ER). **(B)** Calcium Homeostasis: Under stress, SEC61 acts as a passive calcium leak channel; inhibition may help prevent calcium overload and mitochondrial dysfunction, however, calcium effects are likely context dependent **(C)** Antigen Cross-Presentation: SEC61 mediates the retrotranslocation of antigens to the cytosol for proteasomal degradation and subsequent MHC class I presentation to CD8^+^ T cells, although the precise mechanism remains debated. **(D)** Immune Cell Secretion: In activated T cells, SEC61 is required for the biogenesis of pro-inflammatory cytokines (e.g., IL-2, TNF-α, IFN-γ). **(E)** Leukocyte Trafficking: Inhibition reduces the expression of endothelial adhesion molecules and leukocyte surface receptors, limiting leukocyte infiltration into the graft. However, depletion of endothelial junctional proteins may also increase vascular permeability. **(F)** Viral Selectivity: SEC61 inhibition selectively blocks the replication of enveloped viruses (e.g., *SARS-CoV-2, HIV, Influenza*) that rely on the secretory pathway for glycoprotein biogenesis. Potential activity against CMV remains a mechanistic prediction that requires experimental validation, while sparing non-enveloped viruses such as *polyomavirus BK*, which do not depend on SEC61 for entry or assembly.

Opportunistic viral infections, particularly *CMV* and *BK* virus, are major complications of calcineurin-based immunosuppression in kidney transplantation (35). Unlike calcineurin inhibitors, SEC61-targeted agents can, in principle, directly inhibit replication of many enveloped viruses by blocking glycoprotein biogenesis at the ER membrane. Direct experimental evidence demonstrates potent SEC61 inhibitor activity against multiple enveloped viruses. The SEC61 inhibitor Apratoxin S4 inhibits *SARS-CoV-2* replication by blocking spike protein translocation and viral assembly, and exhibits broad-spectrum activity against *influenza A* and *dengue virus* (36). Ipomoeassin F blocks *SARS-CoV-2* spike and ORF8 protein biogenesis via SEC61 inhibition (37). For *HIV*, *SEC61A1* knockdown significantly reduces surface gp120 expression, and chemical SEC61 inhibition suppresses *HIV-1* replication across multiple strains (38). More broadly, many enveloped viruses rely on SEC61-dependent ER translocation and folding of their envelope glycoproteins (39, 40). For *CMV*, a herpesvirus, encodes envelope glycoproteins (gB, gH, gL) that require ER translocation and folding for proper biogenesis. However, direct experimental evidence for SEC61 inhibitor activity against *CMV* is currently lacking. Inhibition of *CMV* replication by SEC61 blockade remains a mechanistic prediction based on analogy to other enveloped viruses and requires dedicated experimental validation before being considered a therapeutic benefit. In contrast, *BK* virus is a non-enveloped polyomavirus whose capsid proteins do not utilize SEC61 for biogenesis. Instead, polyomaviruses exploit components of the ERAD machinery during cell entry rather than SEC61-dependent translocation for virion assembly (41). Therefore, SEC61 inhibitors are unlikely to exert direct antiviral activity against *BK* virus replication, although reduced overall immunosuppressive burden might indirectly preserve antiviral T cell surveillance. A selective SEC61 inhibitor could thus conceptually provide “dual-action” potential in transplantation: dampening alloimmunity while directly inhibiting replication of experimentally validated enveloped viruses (such as *SARS-CoV-2*, *influenza*, and *HIV*) (36–38) at the level of glycoprotein biogenesis. CMV inhibition remains a mechanistic prediction that will require experimental validation. These convergent effects of SEC61 inhibition on protein translocation, calcium homeostasis, alloantigen presentation, effector cytokine production, leukocyte trafficking, and viral replication are depicted schematically in **Figure 1**.

## Lessons from oncology and SEC61 trials: what can be translated?

Natural SEC61-targeting toxins such as mycolactone, coibamide A and Ipomoeassin F first demonstrated that global SEC61 blockade can powerfully suppress secretion and cell viability (10, 18). However, their strong on-target toxicity and lack of client selectivity made them unsuitable as drugs. Oncology has since provided more encouraging evidence through the development and early clinical testing of selective “multi-client” SEC61 inhibitors. Pauwels et al. (18) reviewed several chemotypes that selectively affect a subset of SEC61 clients while preserving most housekeeping proteins. Rehan et al. (11) showed that signal peptide mimicry can bias SEC61 towards client-selective inhibition. Domenger et al. (42) identified SEC61 as a therapeutic vulnerability in multiple myeloma, where SEC61 inhibition induced apoptosis predominantly in malignant plasma cells with high secretory burden. Most importantly, KZR-261—a systemically administered SEC61 inhibitor—has entered phase 1 trials in patients with advanced solid tumors (14, 15). Early clinical data indicate that intermittent dosing schedules can achieve pharmacodynamic evidence of protein secretion inhibition with manageable toxicity and without clear signals of severe nephrotoxicity thus far. In this first-in-human study, a subset of patients experienced stable disease for several months, including some with stability extending beyond 12 months.

The oncology experience yields several lessons relevant to transplantation: (i) SEC61 can be pharmacologically modulated in humans; (ii) client-selective inhibitors preferentially affect high-burden secretory cells such as plasma cells and activated lymphocytes; and (iii) intermittent regimens improve tolerability and may be preferable to chronic continuous exposure. However, important differences between oncology and transplant populations must be considered: transplant recipients require chronic immunosuppression and are at heightened risk of opportunistic infections (43, 44); oncology dosing regimens are typically intermittent and time-limited, whereas transplant immunosuppression is lifelong (45); and the renal vulnerability of transplant patients (46) may differ from oncology patients with intact native kidneys. These differences necessitate dedicated pharmacokinetic, pharmacodynamic, and safety studies in transplant-specific contexts.

## Tubular preservation and clinical windows

The renal proximal tubule is highly sensitive to disturbances in ER Ca^2+^ homeostasis and UPR activation during ischemia–reperfusion (2). Any strategy involving SEC61 inhibition must therefore respect this vulnerability by avoiding blockade during the acute IRI window and introducing it only after the ER and mitochondria have partially recovered. Within this constraint, a selective SEC61 inhibitor could contribute to tubular preservation in the subacute post-transplant period predominantly through indirect mechanisms. Direct experimental evidence demonstrates that SEC61 inhibition potently suppresses cytokine secretion (IL-2, IFN-γ, TNF-α) and reduces chemokine production by immune cells (11, 25, 28). Although direct evidence on inflammatory infiltrates is not available, the diminished chemokine milieu would be expected to limit leukocyte recruitment to the tissue. Moreover, SEC61 inhibition also decreases CD62L lymphocyte expression (10) and endothelial adhesion molecules (33), which may further impair leukocyte adhesion and entry into inflamed sites. Together, these effects are hypothetically likely to attenuate immune-cell–driven paracrine inflammatory stress on tubular epithelium, as direct evidence demonstrating reduced inflammatory infiltrates or tubular protection following SEC61 inhibition is not yet available. While these indirect mechanisms of tubular protection have experimental support (47–50), direct cytoprotective effects of SEC61 inhibition on tubular epithelial cells *in vivo* have not been specifically evaluated. The possibility of additional direct benefits—through reduced SEC61-dependent secretion of pro-fibrotic mediators by stressed tubular cells themselves, or through attenuated chronic ER stress-induced apoptosis—remains speculative and warrants further investigation. Machine perfusion technologies are increasingly used for kidney preservation (51, 52). They offer a platform for ex vivo graft conditioning, raising the question of whether SEC61 modulators could be introduced into preservation solutions. However, the potential for endothelial and tubular toxicity during *ex vivo* perfusion—particularly given SEC61’s role in maintaining endothelial barrier integrity (34) and tubular ER homeostasis (4–9) —represents a significant concern. Rigorous preclinical studies evaluating both efficacy and safety, including assessments of endothelial viability and tubular injury markers, would be required before translation.

**Figure 2:**
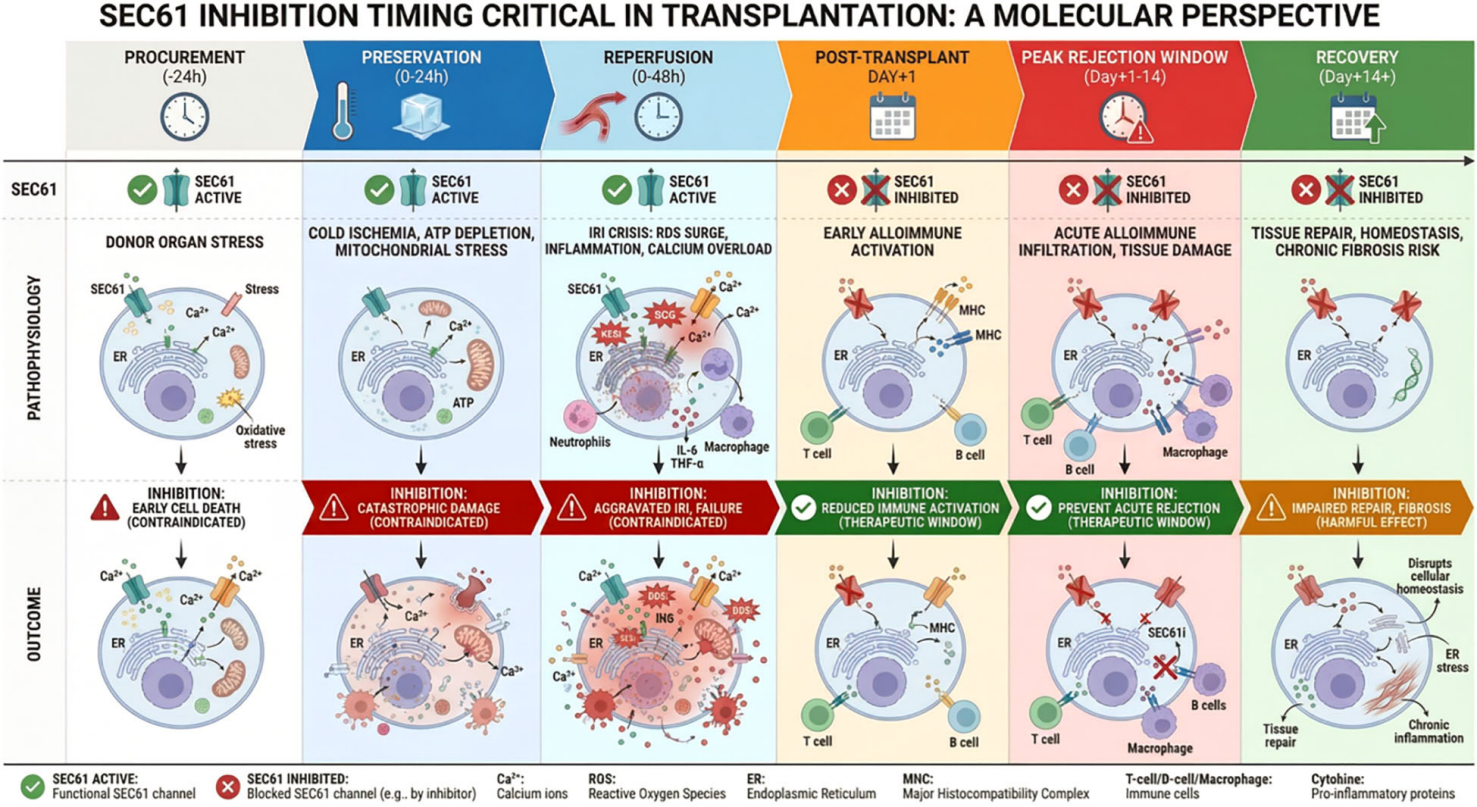
The diagram delineates the pathophysiological phases of kidney transplantation (top) alongside the cellular outcomes of SEC61 inhibition (bottom), defining the optimal potential therapeutic window. This timing strategy is based on mechanistic reasoning and has not been experimentally validated in transplant models. During the procurement to reperfusion period (0–48 h), SEC61 activity is essential for resolving ER stress and restoring calcium homeostasis. Inhibition during this “No-go” zone could aggravate ischemia–reperfusion injury (IRI) and promote early cell death. In contrast, during the subsequent post-transplant period from approximately day +1 to +14, when the alloimmune response intensifies, SEC61 inhibition (SEC61i) may effectively dampen T cell activation and cytokine secretion while preventing acute rejection, representing a potential therapeutic window. This phase could offer a targeted opportunity to modulate immunity without exacerbating initial graft injury. However, in the latter recovery and maintenance phase, (day +14 and beyond), sustained inhibition may interfere with necessary tissue repair mechanisms and fibrosis resolution, suggesting that a transient dosing strategy is preferable to chronic administration.

Based on mechanistic reasoning, the conceptually proposed optimal window for a SEC61 inhibitor would begin after the acute IRI/UPR peak (approximately postoperative day 1) and extend through the first weeks when alloimmune priming is most intense. Discontinuation after the acute rejection-risk window (approximately postoperative day 21–28) would allow restoration of immune competence while maintaining reduced acute rejection risk. This proposed timing strategy and its relation to transplant pathophysiology are summarized in **Figure 2**. Key temporal phases, SEC61-dependent processes, and the anticipated impact of SEC61 inhibition are outlined in **Table 1**.

**Table 1:** Proposed temporal framework for SEC61 inhibition in kidney transplantation.

Phase / time window	Dominant pathophysiology	Key SEC61-dependent processes of interest	Proposed SEC61 inhibitor use	Expected effects / main cautions
Procurement and cold storage	Cold ischemia, energy depletion, early ER stress	Basal ER translocation, adaptive UPR, Ca^2+^ homeostasis	Contraindicated	Risk of impairing adaptive UPR and proteostasis; potential exacerbation of IRI
Reperfusion (0–24 h)	Reoxygenation, ROS burst, peak ER/mitochondrial stress	Resumption of protein synthesis, chaperone induction, ERAD activation	Not recommended (“No-go” zone)	SEC61 needed for chaperone/ERAD synthesis; blockade could tip UPR toward apoptosis
Early post-transplant (Days 1–7)	Alloantigen priming, T cell activation, early rejection risk	Cross-presentation, cytokine secretion, activation receptor expression, viral glycoprotein biogenesis	Preferred therapeutic window (intermittent dosing)	Attenuation of alloimmune priming and effector function; potential antiviral effect; monitor tubular function
Subacute phase (Days 7–28)	Ongoing effector responses, early chronic changes	Sustained cytokine/chemokine secretion, leukocyte trafficking, viral replication	Consider continuation with taper	Further reduction of rejection risk; avoid excessive duration that might impair repair/remodeling
Late maintenance (> Day 28)	Chronic remodeling, repair, infection risk	Homeostatic secretion, tissue repair, adaptive immunity	Generally avoid chronic use	Prolonged blockade may interfere with repair and immune competence; use only in selected scenarios

## Discussion

Taken together, SEC61 has evolved from being regarded as a largely passive conduit for protein import into the endoplasmic reticulum to being recognized as a central regulatory hub linking protein secretion, Ca^2+^ homeostasis, and UPR activation in both the kidney and the immune system. In the setting of kidney transplantation, this unique positioning renders SEC61 simultaneously a vulnerability and a therapeutic opportunity. The same translocon that contributes to tubular ER stress and maladaptive UPR activation during ischemia–reperfusion injury is indispensable for multiple key alloimmune processes, including alloantigen cross-presentation, cytokine synthesis and secretion, activation receptor expression, and leukocyte trafficking, and is further exploited by enveloped viruses for glycoprotein biogenesis and viral assembly. Accordingly, selective and well-timed SEC61 inhibition represents a conceptual framework with possible three-pronged benefit in kidney transplantation by attenuating alloimmune activation, preserving tubular integrity in the subacute phase primarily through indirect anti-inflammatory effects rather than direct cytoprotection, and conferring antiviral activity against experimentally validated enveloped pathogens such as *SARS-CoV-2*, *influenza* and *HIV*. *CMV* inhibition remains a mechanistic prediction based on glycoprotein biology and awaits experimental confirmation. While SEC61 inhibition holds promise by targeting these mechanisms, its broad role in cellular secretion also raises potential risks to graft homeostasis that warrant careful consideration. In kidney transplantation, endothelial cells line the graft vasculature and play a pivotal role in regulating vascular permeability, leukocyte recruitment, and thromboresistance during IRI and alloimmune responses (53)—making them a critical cell type potentially affected by SEC61 inhibition. Thus, the potential for SEC61 inhibition to disrupt endothelial barrier function through depletion of junctional proteins (VE-cadherin, TIE-1/2, JAM-C) represents a safety concern that must be carefully evaluated. Increased vascular permeability could paradoxically exacerbate graft edema and inflammation, and the net balance between beneficial anti-inflammatory effects and potentially harmful vascular effects may be context-dependent and dose-dependent. Importantly, the field has progressed from the use of broadly toxic natural SEC61 inhibitors toward the development of more selective, multi-client small molecules, and early clinical experience in oncology has provided encouraging human safety and tolerability signals, suggesting that pharmacologic modulation of SEC61 is feasible in clinical settings. Nevertheless, several substantial limitations and knowledge gaps must be acknowledged. First, no preclinical studies have yet evaluated SEC61 inhibitors specifically in kidney transplant models, either in rodents or in large animals, and the proposed timing strategy—aimed at exploiting the vulnerability of highly secretory alloimmune and viral processes while respecting adaptive UPR and Ca^2+^ biology in the transplanted kidney—remains a mechanistic hypothesis that requires experimental validation. Second, clinical experience is currently limited to oncology patients, and the safety, tolerability, and immunological consequences of SEC61 inhibition in immunosuppressed transplant recipients are unknown. Third, the long-term effects of chronic or repeated SEC61 modulation on tubular homeostasis, renal function, and adaptive stress responses have not been systematically characterized. Fourth, potential pharmacokinetic and pharmacodynamic interactions between SEC61 inhibitors and standard immunosuppressive agents, as well as their impact on drug exposure and efficacy, have not been studied. Fifth, although SEC61 inhibition has been shown to reduce CD62L expression and suppress IL-2 and IFN-γ production—effects that could theoretically favor tolerance induction or facilitate immunosuppression minimization—this possibility remains speculative and requires dedicated investigation. Sixth, the impact of SEC61 inhibition on regulatory T cells (Tregs) remains unexplored. Tregs are essential for immune tolerance and graft acceptance, relying on both cell-contact mechanisms—such as CTLA-4 and CD39/CD73-mediated adenosine generation—and cytokine-dependent pathways including IL-10 and TGF-β (54, 55). Although Tregs exhibit a comparatively lower secretory burden than activated effector T cells, suggesting they may be proportionally less affected by client-selective SEC61 inhibitors, several Treg-critical surface receptors—including CD25 (IL-2Rα), CTLA-4, and GITR—are type I transmembrane glycoproteins whose biogenesis requires co-translational translocation through the SEC61 channel (4, 10) implying that their expression on Tregs could also be compromised by SEC61 inhibition. Furthermore, SEC61 inhibition potently suppresses IL-2 secretion by effector T cells (10, 11), and since Tregs are critically dependent on paracrine IL-2 for survival, proliferation, and sustained Foxp3 expression (56, 57), reduced IL-2 availability could indirectly compromise Treg homeostasis and function—an effect mechanistically analogous to the well-documented Treg impairment caused by calcineurin inhibitors (58). These considerations reinforce the importance of the transient dosing strategy proposed herein and suggest that complementary approaches, such as low-dose IL-2 supplementation to selectively support Treg survival during SEC61 inhibitor administration, may warrant investigation (59). The net effect of SEC61 inhibition on the Treg/Teff balance in the transplant setting constitutes an important open question that must be addressed in dedicated preclinical models. Consequently, future research should prioritize comprehensive preclinical evaluation of SEC61 inhibitors in kidney transplant models, detailed pharmacokinetic and pharmacodynamic studies defining renal tissue exposure and on-target activity, combination studies with established immunosuppressants to delineate synergy, additivity, or antagonism, development of robust biomarkers to monitor SEC61 target engagement and client-selective inhibition *in vivo*, and systematic exploration of whether SEC61 modulation can contribute to operational tolerance or enable safer minimization of conventional immunosuppression.
